# Microduplication of 16p11.2 *locus* Potentiates Hypertrophic Obesity in Association with Imbalanced Triglyceride Metabolism in White Adipose Tissue

**DOI:** 10.1002/mnfr.202100241

**Published:** 2022-02-05

**Authors:** Dilong Wang, Qiuyan Mai, Xiuyan Yang, Xinjin Chi, Ruohan Li, Jian Jiang, Liang Luo, Xiaoyi Fang, Peng Yun, Liyang Liang, Guang Yang, Kun Song, Liang Fang, Yun Chen, Ying Zhang, Yulong He, Ningning Li, Yihang Pan

**Affiliations:** ^1^ Tomas Lindahl Nobel Laureate Laboratory Precision Medicine Center The Seventh Affiliated Hospital Sun Yat‐Sen University Shenzhen 518107 China; ^2^ Department of Anesthesiology The Seventh Affiliated Hospital Sun Yat‐Sen University Shenzhen 518107 China; ^3^ Department of Emergency The Seventh Affiliated Hospital Sun Yat‐Sen University Shenzhen 518107 China; ^4^ Department of Pediatric The Seventh Affiliated Hospital Sun Yat‐Sen University Shenzhen 518107 China; ^5^ Department of Endocrinology The Seventh Affiliated Hospital Sun Yat‐Sen University Shenzhen 518107 China; ^6^ The Second Affiliated Hospital of Sun Yat‐Sen University Guangzhou 510120 China; ^7^ Department of Burn and Plastic Surgery Department of Wound Repair Shenzhen Institute of Translational Medicine Shenzhen Second People's Hospital The First Affiliated Hospital of Shenzhen University Health Science Center Shenzhen 518116 China; ^8^ Southern University of Science and Technology Shenzhen 518055 China; ^9^ Center for Digestive Disease The Seventh Affiliated Hospital Sun Yat‐sen University Shenzhen 518107 China

**Keywords:** 16p11.2 microduplication, adipogenesis, lipid droplet, metabolic disorder, obesity

## Abstract

**Scope:**

Copy number variation (CNV) of 16p11.2 is a common genetic factor contributing to the etiology of abnormal weight status, while the underlying mechanism is not fully elucidated yet.

**Methods and Results:**

The 16p11.2 CNV mouse model with microduplication of the *7Slx1b‐Sept1* region *(dp/+)* is evaluated under normal chow conditions. Compared to the wild type littermates (WT), the *dp/+* mice exhibit obvious obese phenotype characterized by significant increase in body mass index, fat pad mass, and fat ratio, with visceral‐dominant fat deposits at 12‐week age. White adipose tissue (WAT), liver tissue, and plasma are sampled to assess the comorbid metabolic syndrome. In *dp/+* mice, histopathologic analyses reveal hypertrophic adipocytes and hepatic steatosis; serological examinations show hyperlipemia and hyperinsulinemia. Further, by comparing lipidomic and transcriptomic profiling of epididymal WAT between *dp/+* and WT mice, the study finds the triglyceride (TG) accumulation in *dp/+* mice in association with the dysfunction of lipid droplets. Validation of TG‐metabolism‐associated genes in WAT and in primary cultured adipocytes show enhanced TG synthesis and declined TG hydrolysis in the *dp/+* model.

**Conclusion:**

This study elucidates that the imbalanced TG synthesis/hydrolysis in adipocytic lipid droplets may contribute to the hypertrophic obesity and metabolic disorders in mice with 16p11.2 microduplication.

## Graphical abstract

In this study, a mouse model of 16p11.2 syndrome, carrying microduplication of *Slx1b*‐*Sept1* region, exhibits hypertrophic obesity and metabolic syndrome. Turbulence in lipid composition and gene expression in white adipose tissue suggest a 16p11.2‐rearrangement‐induced impairment of triglyceride hydrolysis in lipid droplets of adipocytes, providing new evidence on how genes in 16p11.2 locus regulate body weight and metabolic status.

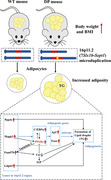

## 1 Introduction

Autism spectrum disorder (ASD) is a set of heterogeneous neurodevelopmental conditions, characterized by restricted repetitive behaviors, impaired communication, and limited social interaction.^[^ ^1^ ^]^ Accumulated evidence indicates that ASD individuals obtain a higher comorbidity burden than the general population, including higher rates of obesity, seizures, and gastrointestinal disorders.^[^ ^2^ ^]^ Indeed, approximately 22.2% of autistic children are diagnosed with obesity.^[^ ^3^ ^]^ Abnormal behaviors in ASD, such as monophagism and decreased physical activity, may contribute to the aberrant weight status,^[^ ^4^, ^5^ ^]^ which in turn exacerbates the ASD core symptoms.^[^ ^6^ ^]^ Therefore, specialized intervention strategies for weight management for ASD related obesity, rather than for common obesity, are thought to be essential to mitigate the autistic symptomatology. However, the molecular basis of ASD‐linked obesity is largely unknown, which remains a great challenge for the current diagnostic and therapeutic approaches for ASD.

Copy number variation (CNV) refers to the microduplication (DP) and microdeletion (DF) of DNA fragments larger than 1kb. Both DP and DF of human chromosome 16p11.2 reveal genetic susceptibility of syndromic ASD, known as 16p11.2 syndrome.^[^ ^7^ ^]^ Interestingly, the CNV of 16p11.2 has a typical dose‐dependent effect on body weight.^[^ ^8^ ^]^ It has been reported that patients with 16p11.2 DP are usually underweight, while those with 16p11.2 DF tend to be obese.^[^ ^9^ ^]^ Obesity caused by 16p11.2 CNV generally manifests severe early‐onset overweight defined as syndromic obesity.^[^ ^9^ ^]^ Patients with syndromic obesity were also reported to be vulnerable to their metabolic defects, such as hyperinsulinemia, hypertriglyceridemia, and diabetes.^[^ ^10^ ^]^ Hence, the 16p11.2 *locus* have been considered as a genomic hotspot to investigate the obese phenotype of ASD individuals.

Several mouse models have been generated to recapitulate features of the autistic neuroanatomy and behavior in 16p11.2 syndrome,^[^ ^11^, ^12^, ^13^ ^]^ but the weight status is still largely unknown. Interestingly, mouse models with engineered *Sultal‐Spn* region, homologous to the human 16p11.2 BP4‐BP5 *locus*, showed obesity in DP mice and underweight in DF mice under a high‐fat diet,^[^ ^13^ ^]^ opposite to the conditions in human. However, those DP mice exhibited a declined endocrine level of leptin, which was the presumed cause of obesity in patient with DF of 16p11.2.^[^ ^14^ ^]^ Supporting this, other two similar mouse models with engineered 7*Slx1b*‐*Sept1* or *Coro1a‐Spn* region obtained a small body size in DF mice, although the weight status of the DP mice was not mentioned.^[^ ^11^, ^12^ ^]^ Thus far, DP mice seem to be suitable models for investigating the role of 16p11.2 rearrangement in obesity genesis, which eclipses clinical case studies where heterogeneity is very high.

The traits of the 16p11.2 syndrome have disclosed the role of white adipose tissue (WAT) in the pathogenesis of the obese phenotype. WAT is an important regulatory organ secreting a series of hormones, such as leptin and adiponectin, for maintenance of metabolic homeostasis in both obese and non‐obese individuals.^[^ ^15^ ^]^ Recent studies reported that dysfunction of WAT was closely related to aberrant lipid dynamics, including dysregulation in storage, synthesis, and mobilization of lipid contents in adipocytes.^[^ ^16^ ^]^ Further, several genes in the 16p11.2 *locus*, such as *Mapk3*, *Gdpd3*, *Ppp4c*, and *Fam57b*, were already found to be involved in the lipid metabolism of adipocytes.^[^ ^17^, ^18^, ^19^, ^20^ ^]^ Therefore, we propose to elucidate the functional changes of WAT in the mouse model of 7*Slx1b*‐*Sept1* DP (*dp/+*), which might achieve a better understanding of the genetic mechanism underlying the genesis and development of obesity in ASD.

Via histological analysis, serological examinations and lipidomics, we determined hypertrophic obesity and metabolic syndrome in *dp/+* mice along with triglyceride (TG) accumulation in WAT under a normal chow condition. Further, transcriptomics and real‐time fluorescence quantitative polymerase chain reaction (RT‐qPCR) validation demonstrated an enhanced TG synthesis but a declined TG hydrolysis in WAT. These findings provide new evidence that the 16p11.2 rearrangement‐induced dysfunction of WAT, particularly in imbalanced TG metabolism, is associated with the abnormal weight status in 16p11.2 syndrome.

## 2 Results

### 2.1 The *dp/+* Mice Exhibit Genetic Obesity with Visceral‐Dominant Fat Deposition and Hypertrophic Adipocytes

**Figure 1:**
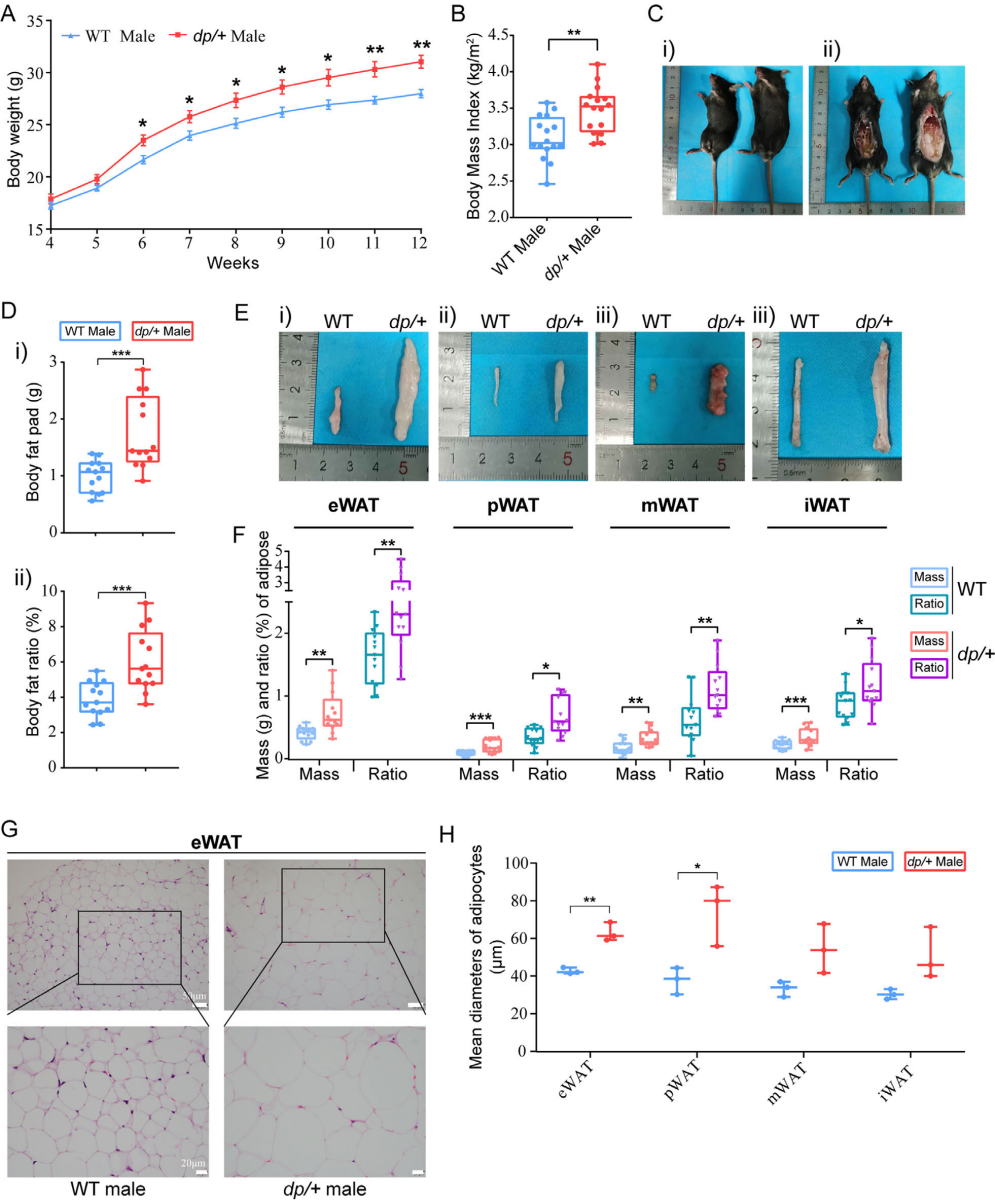
The *dp/+* mice exhibit an obesity phenotype with visceral‐dominant fat deposition and hypertrophic adipocytes. A) The curve for weight record of male mice from 4th to 12th week after birth and statistical analysis, WT *n* = 8, *dp/+ n* = 8. B) Statistical diagram for body mass index of male mice at 12‐week age, WT (*n* = 16) and *dp/+* (*n* = 16). C) Representative images of male 12‐week‐old WT (left) and *dp/+* (right) mice. D) Statistical diagram of fat pad mass (g) and fat ratio (%) of male 12‐week‐old mice, WT *n* = 13, *dp/+ n* = 13. E) Representative images of eWAT, pWAT, mWAT, and iWAT from male WT (left) and *dp/+* (right) mice at 12 weeks old. F) Statistical diagram for adipose mass and ratio of eWAT, pWAT, iWAT, and mWAT from male 12‐week‐old mice, WT *n* = 13, *dp/+ n* = 13. G) Representative H&E images of eWAT for male WT and *dp/+* mice at 12‐week‐old (scale bars, 50, 20 µm). H) Statistical diagram for mean adipocytes diameters (µm) based on H&E staining images of eWAT, pWAT, mWAT, and iWAT, WT *n* = 3, *dp/+ n* = 3. All data were presented as boxplots with whisker indicating the sample minimum to sample maximum through all the quartiles. Significance was evaluated by unpaired *t*‐test between WT and *dp/+*, with *p < 0.05, **p < 0.01, ***p < 0.001, ****p < 0.0001. eWAT indicates epididymal white adipose tissue; iWAT, inguinal white adipose tissue; mWAT, mesenteric white adipose tissue; pWAT, perirenal white adipose tissue; WAT, white adipose tissue.

To determine whether the *dp/+* model could recapitulate the obese phenotype in a normal chow condition, we set about monitoring the body weight of male mice weekly from 4th to 12th week of their age. Compared to their gender‐matched wild type (WT) littermates, male *dp/+* mice already displayed significant overweight at 6‐week age, and kept a higher weight gain since then (**Figure** **1**A). At 12‐week age, the male *dp/+* mice exhibited a significantly higher body mass index (BMI) (Figure 1B) and an obviously larger body size (Figure 1C) than the WT controls. In keeping with these observations, the mass of fat pad (g) and the fat ratio (%) were also significantly increased (Figure 1D) in the *dp/+* mice. Although the effects of 16p11.2 CNVs on BMI was previously reported,^[^ ^14^, ^21^, ^22^ ^]^ the distribution of body fat was yet unclear in human or animal studies. Hence, we assessed the visceral adipose tissue (VAT) (e.g., epididymal WAT [eWAT], perirenal WAT [pWAT], and mesenteric WAT [mWAT]) and the subcutaneous adipose tissue (SCAT) (e.g., inguinal WAT [iWAT]) separately (Figure 1E–H). We found that the eWAT, pWAT, and mWAT of the *dp/+* mice significantly increased to 76%, 121%, and 93% in mass, respectively, while the iWAT of the *dp/+* mice significantly increased to 51% in mass, compared to that of the WT controls (Figure 1F, mass). We also found in the body weight of the *dp/+* versus WT mice increased mass proportion of both VAT and SCAT, where the eWAT accounted for the highest proportion (Figure 1F, ratio). Further, via H&E staining (Figure 1G and Figure S2, Supporting Information), we observed significantly enlarged size of adipocytes in these adipose tissues of *dp/+* mice (Figure 1H), indicative of a typical pathological feature of hypertrophic obesity. Collectively, our data revealed a genetically induced hypertrophic obesity characterized by overweight, visceral‐dominant fat deposition, and hypertrophic adipocytes in the *dp/+* mice.

Interestingly, there were no significant differences in both food and water intake between *dp/+* and WT mice during the entire observation period (Figure S3A, Supporting Information). The 12‐week‐old *dp/+* mice showed unchanged traveling distance and speed in the open field test (Figure S3B, Supporting Information), suggesting a normal spontaneous movement. It has been reported that the gender was also an important confounder affecting weight status due to sex hormone regulations.^[^ ^23^, ^24^ ^]^ Thus, we measured body weight, BMI, fat mass, and adipocyte size in female *dp/+* mice versus age‐matched WT littermates. Supporting our finding in males, the female *dp/+* mice also showed a hypertrophic obese phenotype (Figure S4, Supporting Information). These data demonstrated that the abnormal weight gain of *dp/+* mice was genetically induced by DP of the *7Slx1b‐Sept1* region, rather than by other factors, such as diet, movement activity, sex chromosome, or sex hormones.

### 2.2 DP of *7Slx1b‐Sept1* Region Gives Rise to Metabolic Syndrome in Mice

#### 2.2.1 Hyperlipidemia and Hyperinsulinemia in *dp/+* Mice

**Figure 2:**
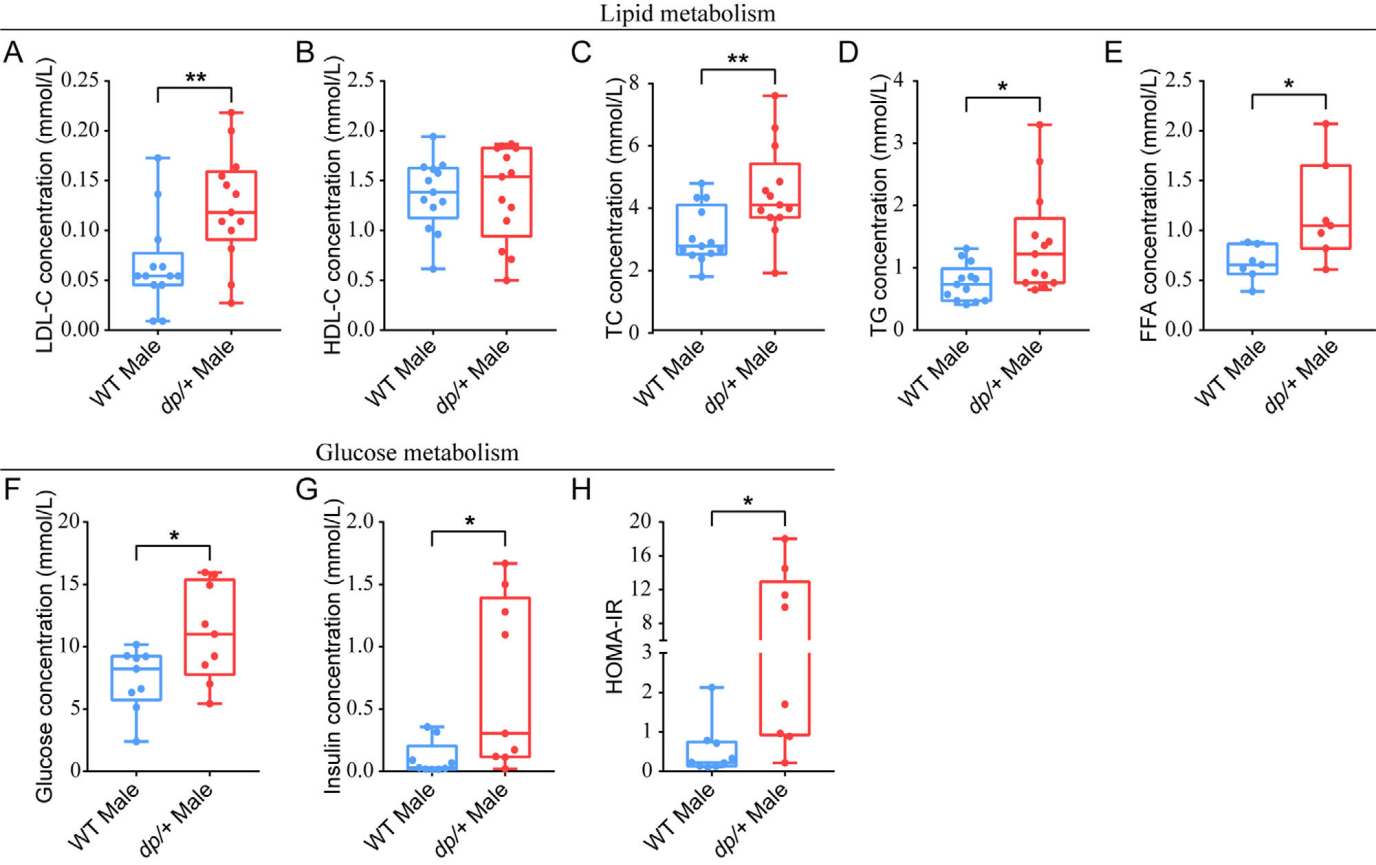
Hyperlipidemia and hyperinsulinemia in *dp/+* mice. Statistical diagrams for lipid metabolism associated indexes at 12‐week‐old. A) Concentrations of LDL cholesterol (mmol L^−1^) in the plasma of *dp/+* (*n* = 13) and WT mice (*n* = 13). B) Concentrations of HDL cholesterol (mmol L^−1^) in the plasma of *dp/+* (*n* = 13) and WT mice (*n* = 13). C) Concentrations of total cholesterol (mmol L^−1^) in the plasma of male *dp/+* (*n* = 13) and WT mice (*n* = 13). D) Concentrations of triglyceride (mmol L^−1^) in the plasma of male *dp/+* (*n* = 13) and WT mice (*n* = 13). E) Concentrations of free fatty acids (mmol L^−1^) in the plasma of male *dp/+* (*n* = 7) and WT mice (*n* = 7). Statistical diagrams for glucose metabolism associated indexes at 12‐week‐old. F) Fasting glucose (mmol L^−1^) levels of male *dp/+* (*n* = 9) and WT mice (*n* = 9). G) Insulin (mmol L^−1^) levels of male DP (*n* = 9) and WT mice (*n* = 9). H) Homeostatic model assessment of insulin resistance (HOMA‐IR) of male *dp/+* (*n* = 9) and WT mice (*n* = 9). All data were presented as boxplots with whisker indicating the sample minimum to sample maximum through all the quartiles. Significance was evaluated by unpaired *t*‐test between WT and *dp/+*, with *p < 0.05, **p < 0.01.

Since both visceral fat deposition and enlarged adipocyte size are linked to metabolic disorders in obese individuals,^[^ ^25^, ^26^, ^27^ ^]^ we asked whether the *dp/+* mice manifested metabolic complications. We measured at the serous level a series of indexes associating to lipid and glucose metabolism in the *dp/+* mice versus WT controls at 12‐week age. We found that the *dp/+* mice displayed significantly increased levels of low‐density lipoprotein cholesterol (LDL‐C), total cholesterol (TC), triglyceride (TG), and free fatty acids (FFA), while high‐density lipoprotein cholesterol (HDL‐C) remained unchanged (**Figure** **2**A–D), suggesting hyperlipidemia and impaired lipid metabolism. Meanwhile, the levels of glucose, insulin, and homeostatic model assessment of insulin resistance (HOMA‐IR) were significantly increased (Figure 2F–H), suggesting hyperinsulinemia and insulin resistance. As two of the most profound complications of obesity, hyperlipidemia and hyperinsulinemia are also closely related with the morbidity of cardiovascular diseases and type 2 diabetes mellitus.^[^ ^28^ ^]^

#### 2.2.2 Ectopic Lipid Accumulation in *dp/+* Mice

**Figure 3:**
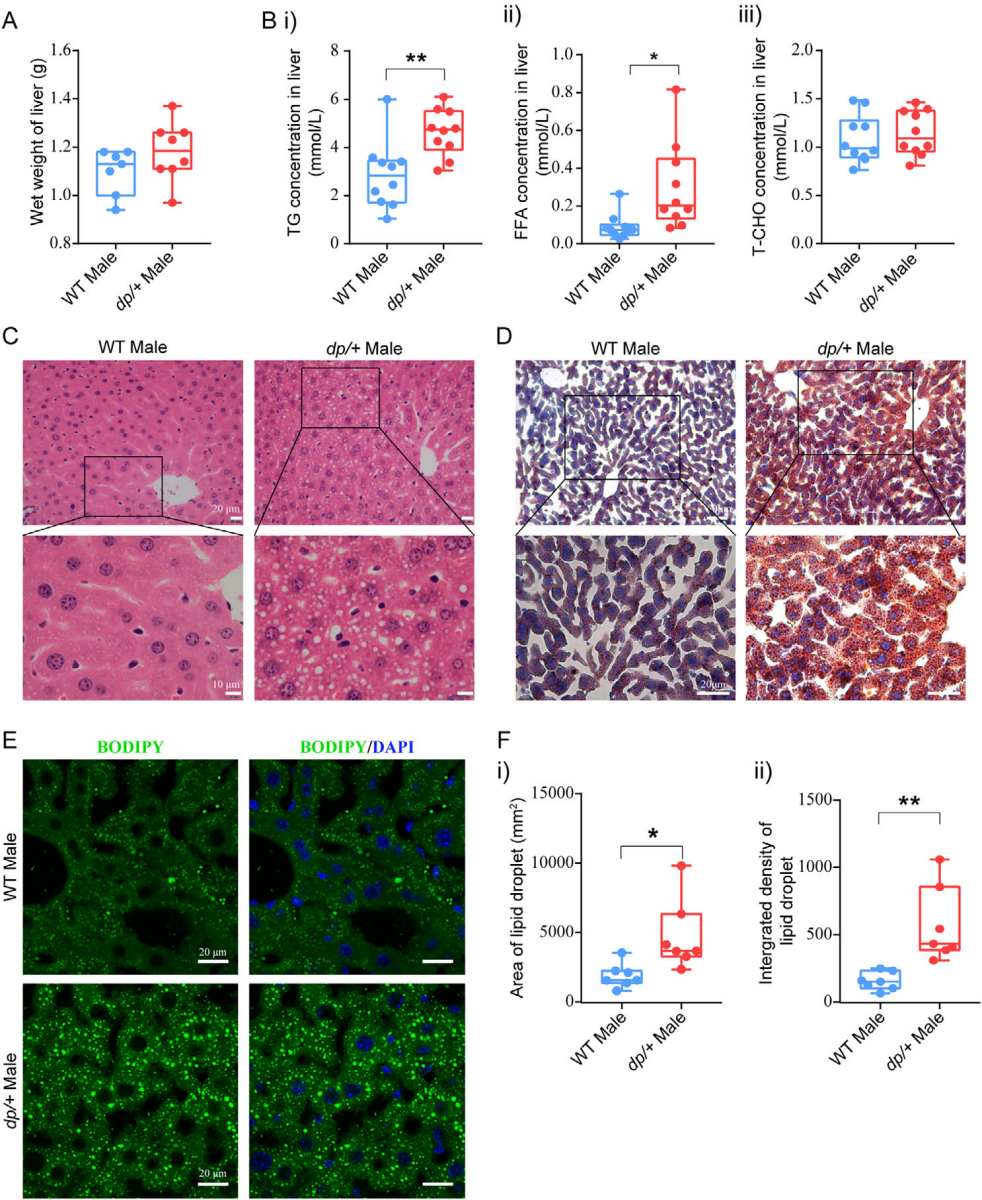
Ectopic fat deposition in liver of *dp/+* mice. A) Statistical diagram of wet liver weight of male mice at 12‐week‐old, WT (*n* = 7) and *dp/+* (*n* = 8). B) Statistical diagrams of lipid metabolism associated indexes in liver of male mice at 12‐week‐old: concentration of i) triglyceride (mmol L^−1^), ii) free fatty acids (mmol L^−1^), and iii) total cholesterol (mmol L^−1^), WT (*n* = 10), *dp/+* (*n* = 10). C) Representative H&E images of livers for male WT and *dp/+* mice at 12 weeks. (Scale bars, 20, 10 µm). D) Representative Sudan III images of livers for male WT and *dp/+* mice at 12 weeks. (Scale bars, 50, 20 µm). E) Representative BODIPY (493/503) images of livers for male WT and *dp/+* mice at 12 weeks. (Scale bars, 20 µm). F) Statistical diagrams of area and integrated density of lipid droplets in the livers of male 12‐week‐old WT (n = 7) and *dp/+* mice (n = 7). All data were presented as boxplots with whisker indicating the sample minimum to sample maximum through all the quartiles. Significance was evaluated by unpaired *t*‐test between WT and *dp/+*, with *p < 0.05, **p < 0.01.

Considering that insulin resistance and pertinent metabolic disturbances are associated to ectopic fat accumulation, and the excess lipids are stored in metabolic organs such as liver and muscle, we sought to investigate the body burden resulted from the aforementioned metabolic disorders in the *dp/+* mice. We first assayed whether the DP of *7Slx1b‐Sept1* region could cause pathophysiological changes in liver. We found that at 12‐week age the wet weight of liver in *dp/+* mice was slightly increased than that in the WT controls (**Figure** **3**A). Further, via quantifying lipid associated indexes, we found significantly increased levels of hepatic TG and FFA, and unchanged level of TC in the *dp/+* mice (Figure 3B). Since fat could be stored and hydrolyzed in the form of TG and FFA respectively, the simultaneous increment of TG and FFA in liver implied overloaded TG synthesis and impaired fat storage in the *dp/+* mice.

According to our H&E staining on the liver slices, we observed obvious steatosis in the dp/+ mice characterized by diffused microvacuole (Figure 3C). Indeed, the Sudan III staining confirmed massive lipid accumulation in hepatic lobules (Figure 3D). Moreover, by utilizing fluorescent dye BODIPY (493/503), the standard dye to detect lipid droplets, we found enhanced lipid droplet formation in hepatocytes (Figure 3E,F). In addition to the liver, we also observed significantly increased TG concentration in the muscles of *dp/+* mice (Table S1, Supporting Information). These data suggested a global ectopic fat deposition induced by DP of the *7Slx1b‐Sept1* region.

### 2.3 TG Accumulation Features the Lipid Composition Alteration in eWAT of *dp/+* Mice

**Figure 4:**
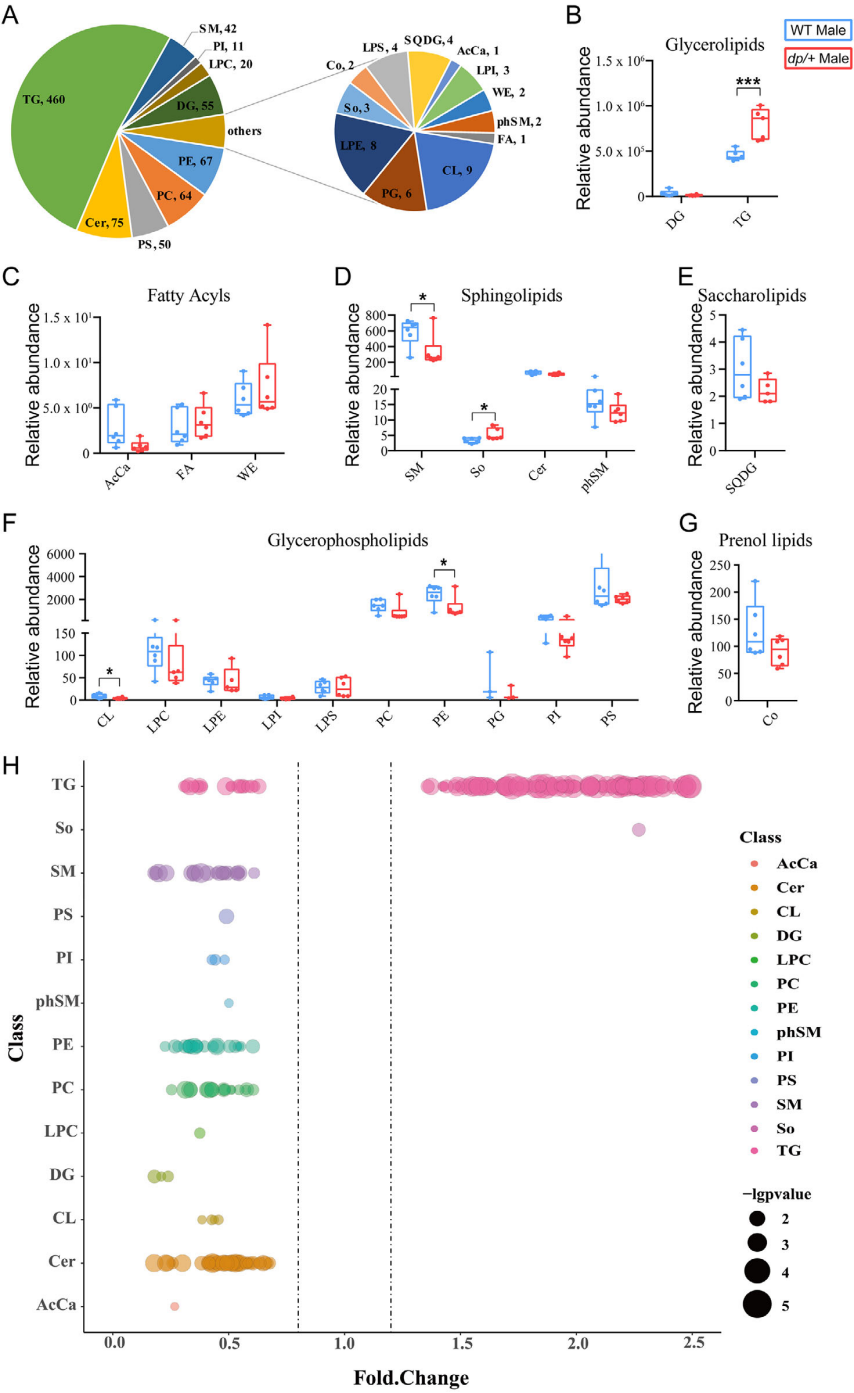
Lipidomics of eWAT reveals an altered lipid composition particularly in TG levels in *dp/+* mice. A) Distribution of lipid classes in eWAT samples of WT male (*n* = 6) and *dp/+* mice (*n* = 6) was evaluated by lipidomics. Statistical diagrams of the relative abundance of lipid classes in glycerolipids B), fatty acyls C), sphingolipids D), saccharolipids E), glycerophospholipids F), and prenol lipids G). H) Bubble diagram of differential lipid species (*p*  <  0.05) in in male *dp/+* versus WT mice. Each dot represents a lipid species, and the dot size indicates significance. All data were presented as boxplots with whisker indicating the sample minimum to sample maximum through all the quartiles. Significance was evaluated by unpaired t‐test between WT and *dp/+*, with *p < 0.05, ***p < 0.001.

To determine the possible changes of the lipid composition in response to DP of the *7Slx1b‐Sept1* region, we isolated eWAT, where fat is dominantly deposited in the *dp/+* mice, for lipidomic profiling in 12‐week‐old mice. We identified a total of 889 different lipid species between *dp/+* and WT mice, consisting of 460 in the TG class, 75 in Ceramide, 67 in phosphatidylethanolamine, 64 in phosphatidylcholine, 55 in diglyceride, 50 in phosphatidylserine, and 45 in other lipid classes (**Figure** **4**A). At the class level, we found that TG in glycerolipids (Figure 4B) and sphingosine in sphingolipids (Figure 4D) were both significantly increased, while acyl carnitine in fatty acyls (Figure 4C), sphingomyelin in sphingolipids (Figure 4D), cardiolipin in glycerophospholipids, and phosphatidylethanolamine in glycerophospholipids (Figure 4F) were significantly decreased in *dp/+* versus WT mice. Next, we utilized the bubble map to visualize the differential lipid species, with *p* value of 0.05 as a cutoff. We found that most species in the TG class were increased, in sharp contrast to the profound decline in most other lipid species in the *dp/+* mice (Figure 4H). Our data suggested that the DP of the *7Slx1b‐Sept1* region resulted in considerable alterations in adipose lipid compositions, particularly in the TG accumulation, constituting a metabolic signature underlying the obese phenotype of the *dp/+* mice.

### 2.4 DP of *7Slx1b‐Sept1* Region Induces an Imbalance Between Synthesis and Hydrolysis of TG in WAT of Mice

**Figure 5:**
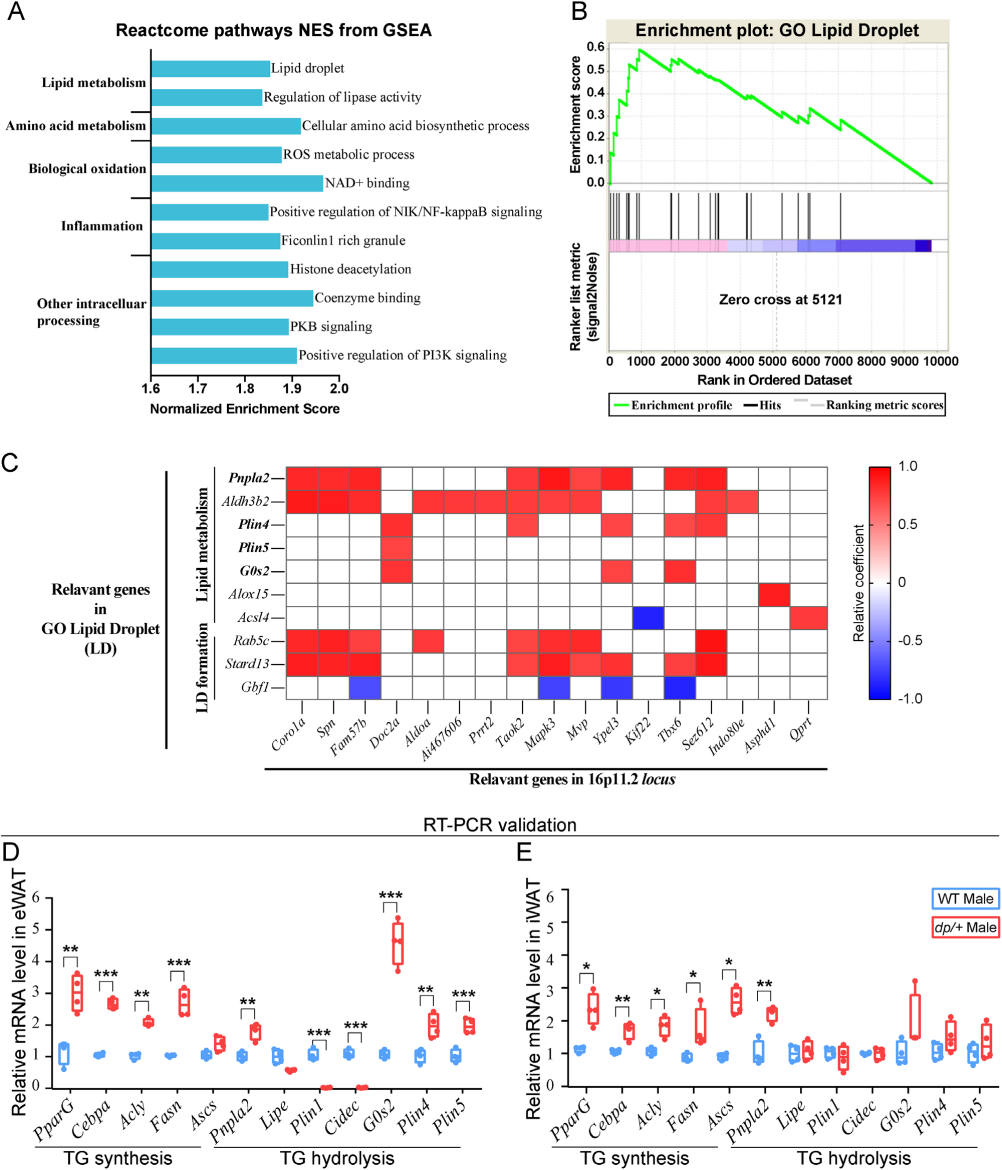
Transcriptomic analysis elaborates an abnormal lipid‐droplet‐associated TG metabolism in WAT of *dp/+* mice. Transcriptomis was peformed with eWAT from male 12‐week‐old WT (*n* = 4) and *dp/+* mice (*n* = 4). A) Reactcome pathway normalized enrichment scores (NES) of differential pathways in *dp/+* versus WT mice according to Gene Set Enrichment Analysis (GSEA). B) GSEA enrichment map of lipid droplet pathway. C) Correlation matrix between the expression levels of lipid‐droplet‐pathway‐related genes and the expression levels of 16p11.2 locus genes. Red and blue panes indicated significant positive and negative correlation (*p* < 0.05) respectively; white panes indicated no correlation (*p* ≥ 0.05). Subsequent validation for genes involving in triglyceride (TG) metabolism was performed via RT‐PCR on eWAT and iWAT, WT *n* = 4, *dp/+ n* = 4. Statistical diagrams for the expression level of genes involving TG metabolism in eWAT D) and iWAT E). Significance was evaluated by unpaired *t* test between WT and *dp/+*, with *p < 0.05, **p < 0.01, ***p < 0.001. RT‐qPCR indicates real‐time fluorescence quantitative PCR.

It is generally accepted that intracellular TG accumulation accounts for the expansion of adipocytes and acts as a hallmark of obesity. To further investigate the molecular mechanism underlying the TG accumulation in the *dp/+* mice, we sampled eWAT from 12‐week‐old mice for transcriptomic analysis. The gene expression profiling showed a total of 1431 up‐regulated genes and 1184 down‐regulated genes in eWAT in *dp/+* versus WT mice. For functional analysis, we exploited the Gene Set Enrichment Analysis (GSEA) and identified 11 differential pathways, highlighting abnormalities in lipid and amino acid metabolism, biological oxidation, and inflammation in the *dp/+* mice (**Figure** **5**A). Of note, the pathway of lipid droplets was significantly altered (Figure 5B), in congruence with our lipidomic findings of accumulating TG, since lipid droplets are the ultimate intracellular storage venue for TG in WAT. Via Pearson analysis, we found the expression levels of genes in the lipid droplet pathway were significantly correlated with the expression levels of genes within the *7Slx1b‐Sept1* region, suggesting that the lipid metabolism and lipid droplet formation are most likely to be regulated by DP of *7Slx1b‐Sept1* region (Figure 5C).

To tease out the essential molecular regulators of the TG accumulation in the *dp/+* mice, we reached out to literature^[^ ^29^, ^30^, ^31^ ^]^ and set up a collection of TG metabolism‐associated genes in WAT, including *Pnpla2*, *Plin4*, *Plin5*, and *G0s2*, the four genes involved in the hydrolysis of TG in lipid droplets as identified above in the lipid droplet pathway. It is worth noting that *Pnpla2* and *lipe* encode the two key lipases (adipose triglyceride lipase [ATGL] and hormone‐sensitive lipase [HSL]) that sequentially hydrolyze TG and diglyceride respectively, while *Plin5*‐ and *Gos2*‐encoded proteins protect TG from being hydrolyzed. We performed RT‐qPCR on eWAT and iWAT from 12‐week‐old mice to validate the expression levels of these regulatory genes. Intriguingly, we found an enhanced TG synthesis in eWAT in *dp/+* versus WT mice, characterized by significantly increased *PparG*, *Cebpa*, *Acly*, and *Fasn*, that are involved in *de novo* synthesis of TG from glucose; on the contrary, the TG hydrolysis in eWAT of the *dp/+* mice was impaired, mainly characterized by significant increment of *G0s2*, *Plin4*, and *Plin5* (Figure 5D). Unexpectedly, *Pnpla2* was significantly increased and *Lipe* was nominally decreased without significance in *dp/+* mice. Although these two genes encode the key lipases, the enzyme activities are regulated by master regulons on lipid droplets surface, such as Perilipin (PLIN) family.^[^ ^32^ ^]^ The imbalanced TG metabolism that we consolidated in the *dp/+* mice strongly indicated an inefficient TG renew in adipocytes, which is usually considered as a cause for expansion of adipocytes^[^ ^33^ ^]^ and is also in support of the hypertrophic adipocytes in the context of our study. In iWAT, while the TG synthesis was enhanced, the TG hydrolysis was not altered in the *dp/+* mice (Figure 5E), suggesting disturbed TG metabolism in iWAT but no worse than that in eWAT, in line with the phenotype of visceral‐dominant fat deposition we found in the *dp/+* mice. Considering that the liver could influence lipid metabolism via regulating cholesterol homeostasis,^[^ ^34^ ^]^ we also checked a collection of cholesterol metabolism‐related genes in liver by RT‐qPCR. Most of these genes were not significantly changed in liver of *dp/+* mice (Figure S5, Supporting Information), suggesting that cholesterol metabolism in the liver is less likely to be involved in the pathogenesis of metabolic syndrome in our DP model. Taken together, our findings demonstrated a robust relationship between the DP of the *7Slx1b‐Sept1* region and the imbalanced TG metabolism in WAT, which may account for the etiology of hypertrophic obesity in the *dp/+* mice.

### 2.5 DP of *7Slx1b‐Sept1* Region Potentiates Preadipocyte Differentiation and Lipid Droplet Formation in Association with Imbalanced TG Metabolism

**Figure 6:**
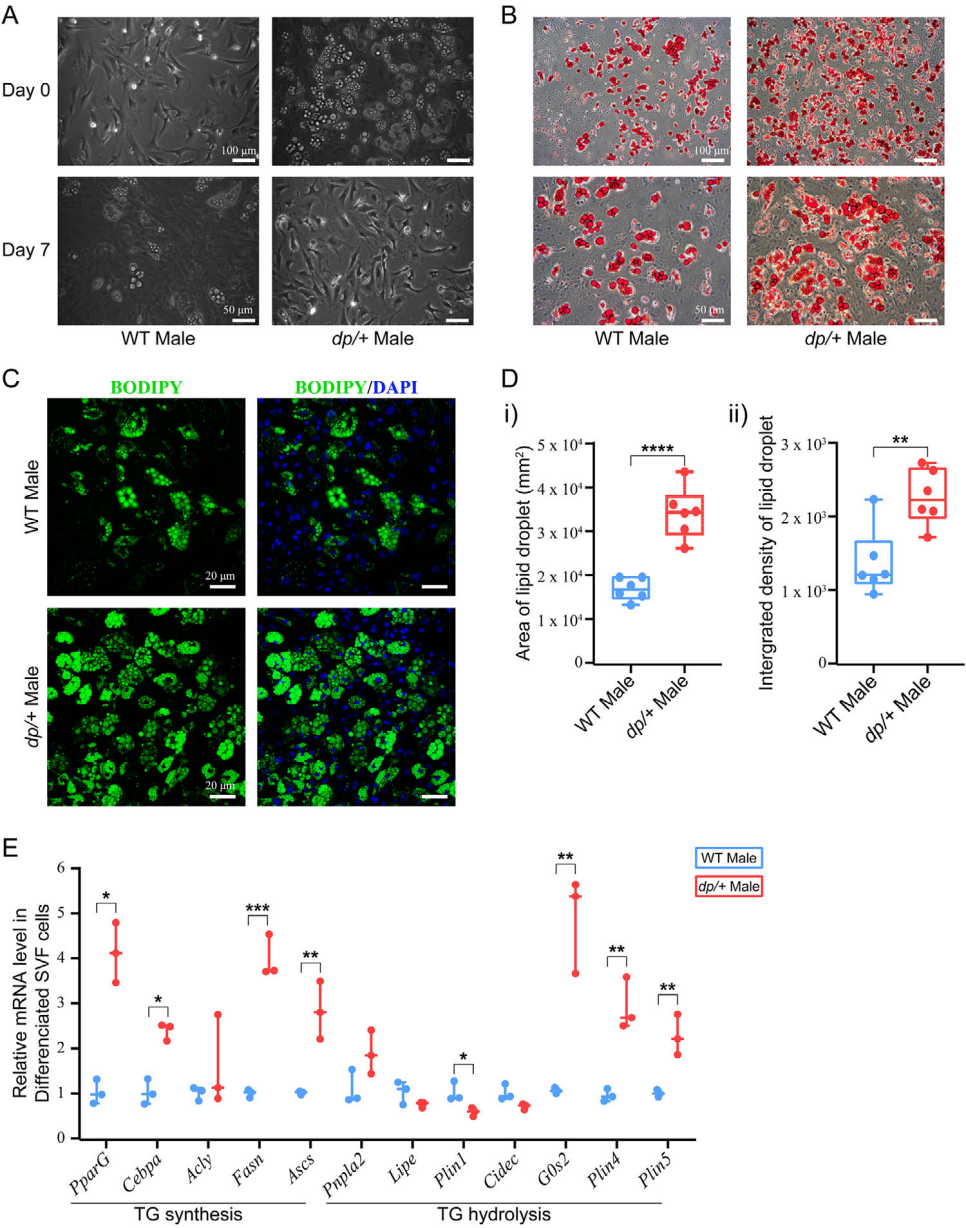
DP of 16p11.2 induces abnormal lipid droplet formation via affecting TG metabolism. SVF cells was isolated from WT and *dp/+* mice for the differentiation experiment. A) Typical morphology of SVF cells before and 7 days after induction (Scale bars, 50 µm). B) Representative images of Oil Red O for differential SVF cells at the 7th day after induction (Scale bars, 100; 50 µm). C) Representative images of BODIPY (493/503) staining for differential SVF cells at the 7th day after induction (Scale bars, 20 µm). D) Statistical diagrams of i) area and ii) integrated density of lipid droplets in differential SVF cells based on BODIPY (493/503) staining images, WT *n* = 6, *dp/+ n* = 6. E) Statistical diagram for expression level of the genes involving in TG metabolism, WT *n* = 3, *dp/+ n* = 3. All data were presented as boxplots with whisker indicating the sample minimum to sample maximum through all the quartiles. Significance was evaluated by unpaired *t*‐test between WT and *dp/+*, with *p < 0.05, ***p < 0.001.

In order to corroborate the direct role of 16p11.2 DP in metabolic changes of adipocytes, we isolated stromal vessel fraction (SVF) cells, the adipocyte precursors, from iWAT of both *dp/+* and WT mice, and performed in vitro differentiation experiments (**Figure** **6**A). After 7 days of induction of differentiation, we utilized Oil Red O staining to assess the differentiation of SVF cells. Clear signal of lipid droplets in both *dp/+* and WT adipocytes were shown (Figure 6B). Further, we performed BODIPY (493/503) staining to accurately measure the area and intensity of lipid droplets signal (Figure 6C). The *dp/+* adipocytes showed significant enhancement of both area and integrated density of lipid droplets than WT adipocytes, suggesting a progressive pathophysiological state of metabolic syndrome and perhaps a faster trajectory leading to differentiation and lipid droplet formation in the *dp/+* mice. Next, we collected the adipocytes to validate the aforementioned genes in the TG metabolism via RT‐qPCR. As shown in Figure 6D, we confirmed an enhanced TG synthesis and a weakened TG hydrolysis in the *dp/+* adipocytes, which was consistent with our in vivo findings in the *dp/+* mice. Therefore, dysregulation of TG‐metabolism‐associated genes in adipocytes might play a key role in accelerating differentiation and lipid droplet formation of adipocytes in *dp/+* mice.

## 3 Discussion

This study identified a genetically induced obese phenotype, characterized by visceral‐dominant fat deposit, hypertrophic adipocytes, and comorbidity of metabolic syndrome (e.g., hyperlipidemia, hyperinsulinemia, and ectopic lipid deposition) in the *dp/+* mice, a well‐known CNV model of ASD (Figures 1, 2, 3). Lipidomics revealed overall altered lipid composition characterized by TG accumulation in eWAT (Figures 4). In line with this, transcriptomics elaborated dysfunction of lipid droplets, which was associated with the overexpression of 16p11.2 genes. Notably, via the comprehensive validation of TG‐metabolism‐associated genes, we found enhanced synthesis and declined hydrolysis of TG in eWAT of the *dp/+* mice (Figures 5). The in vitro experiments consolidated that the DP of the *7Slx1b‐Sept1* region could accelerate the adipocytes differentiation and lipid droplets formation in association with the imbalanced TG metabolism (Figure 6).

We identified the impaired TG hydrolysis in WAT as being responsible for the TG accumulation and adipocytes expansion in the *dp/+* mice. Disturbance of TG hydrolysis in adipocytes has long been recognized to be associated with metabolic disorders.^[^ ^30^ ^]^ Here, we observed metabolic syndrome, consisting of hyperlipidemia, hyperinsulinemia, ectopic lipid deposition, and adipocytic TG accumulation, in the *dp/+* mice. It is widely accepted that the TG hydrolysis is mediated by the activity of the key lipases, ATGL (i.e., *Pnpla2*) and HSL (i.e., *Lipe*),^[^ ^35^ ^]^ and the decisive control of TG hydrolysis is determined by proteins on the lipid droplets surface, including G0/G1 switch 2 (*G0s2*) and PLIN family (*Plin4* and *Plin5*).^[^ ^32^ ^]^ Of note, several studies reported that the overexpression of G0S2 in human adipocyte substantially diminished lipolysis by disturbing ATGL activity and it's co‐localization to lipid droplets.^[^ ^36^, ^37^ ^]^ Our and these studies supported that the rearrangement of 16p11.2 *locus* may trigger the development of syndromic obesity via causing dysfunction of adipocellular lipid droplets in TG hydrolysis. Besides, in the *dp/+* mice, we also observed a higher expression of peroxisome proliferator‐activated receptor gamma (*PparG*), the key regulon for metabolic feature orchestration specifically in adipocytes.^[^ ^20^, ^38^ ^]^ To figure out whether disturbance of TG metabolism was directly triggered by 16p11.2 DP or indirectly influenced via *PparG* needs further investigation, say, via conditional knock out of *PparG* in the DP model.

Abnormal lipid metabolism has been implicated in both obesity and ASD.^[^ ^39^, ^40^ ^]^ Clinical evidence revealed that maternal obesity with altered lipid profiling was associated with increased prevalence of ASD in offspring,^[^ ^41^ ^]^ suggesting the likelihood that some lipid molecules could lead to the development of ASD. Recently, we reported significant decline in sphingomyelin species and ceramide species (i.e., sphingolipids) in the striatum of the *7Slx1b‐Sept1* DF model of ASD, which was directly linked to abnormal myelin structure.^[^ ^42^ ^]^ In keeping with this, decrease of species in sphingomyelin and ceramide was observed in eWAT of the current DP model of ASD. It is therefore suggested that both DF and DP of the 16p11.2 *locus* were related to ASD due to the convergent role of sphingolipids. However, accumulation of TG (i.e., glycerolipids) found in the DP model was not seen in the DF model. Given the different biological function between glycerolipids and sphingolipids, it is likely that autistic symptoms and obese phenotype in the *dp/+* mice could be resulted from abnormal metabolism of distinct lipids.

Limited clinical evidence suggested that a distal gene in 16p11.2 *locus*, SH2B adaptor protein 1 (SH2B1), was the candidate affecting the weight status in patients with 16p11.2 syndrome, due to its function in regulating leptin sensitivity.^[^ ^43^ ^]^ Arbogast et al^[^ ^13^ ^]^ reported an obese phenotype along with a declined endocrine level of leptin in the *Sultal‐Spn* DP model with high‐fat diet. However, neither the expression of *Sh2b1* nor the level of leptin was changed in our DP model with normal chow (Figure S6, Supporting Information). It is possible that the dysfunction of leptin is susceptible to abnormal dietary habits rather than genetic turbulence. Moreover, our observations disentangled molecular basis of the obese phenotype from confounders including dietary, locomotive and sexual factors, and revealed that the DP of the *7Slx1b‐Sept1* region independently induced an obese phenotype in mice. Indeed, several genes in the 16p11.2 *locus* have been reported to be involved in lipogenesis and adipogenesis. A recent study revealed that overexpression of *glycerophosphodiester phosphodiesterase domain containing 3* (*Gpdp3*) in both human and mouse was associated with hepatic TG accumulation.^[^ ^17^ ^]^ Several in vitro studies pointed out that extracellular signal‐regulated kinase 1/2 (i.e.*, Mapk3*) and *family with sequence similarity 57, member b* (*Fam57b*) could directly interact with *PparG*. Taken together, these results provided new evidence that highlighting the regulatory role of proximal genes within the 16p11.2 *locus* in the pathogenesis of the comorbid obese phenotype in ASD.

## 4 Conclusion

In conclusion, via integrated analyses of histopathology, serology, lipidomics, and transcriptomics in mice with 16p11.2 duplication, we demonstrated that the genetically‐induced synthesis/hydrolysis imbalance of TG in adipocytic lipid droplets may constitute a critical molecular program in the development of obesity and metabolic syndrome under the overarching psychiatric disorders with 16p11.2 DP. These findings may shed light on new therapeutic strategies and treatment paradigms that could be incorporated to reduce the metabolic complications of ASD in children.

## 5 Experimental Section

### Mice

The 7*Slx1b*‐*Sept1* DP mice were purchased from the Jackson Laboratory (#01 3129) with a C57BL/6J genetic background. The mice carried a heterozygous DP of an approximately 0.44 Mb DNA fragment in Chromosome 7, which was homologous to the human 16p11.2 *locus* (Figure S1A, Supporting Information). The male *dp/+* mice were crossed with female C57BL/6J WT mice to obtain heterozygous and WT genotype (Figure S1B,C, Supporting Information). In this study, 16 male *dp/+* mice and 12 female *dp/+* mice were used, along with their gender‐matched WT littermate as controls. The experimental mice were housed in a pathogen‐free facility under 12‐h light/12‐h dark cycle and separated after weaning according to their genotype and gender. All protocols were approved by the Animal Ethics Committee of Sun Yat‐Sen University (SYSU‐IACUC‐2021000056).

### Body Weight, Food and Water Intake, and Fat Composition

Body weight was measured weekly on normal chow diet from 4‐week‐old to 12‐week‐old. For food and water intake measurement, mice were housed separately in individual cage. Each cage was supplied three times a week with fodder and water in equal. Remaining status of feed of each cage was record at every rotation, and the average weekly consumption of food and water were calculated. The BMI was calculated at 12‐week‐old, formulated by dividing body weight (g) by body length (mm) square. For fat composition measurement, the mice were sacrificed via cervical dislocation. Different types of WAT, including eWAT, pWAT, mWAT, and iWAT, were dissected and weighted. Fat mass (g) and fat ratio (fat mass/body weight) of WAT were calculated.

### Lipid Profile Determination and Endocrinological Analysis

Mice fasted for 12 h were subjected to sample plasma and liver tissue. Heparin sodium anticoagulation tubes (Jetway, China, GSN KNG) were used to collect orbital venous blood from mice, and then were centrifuged at 3500 rpm for 10 min to obtain the plasma for subsequent arrays. Liver tissue was dissected form mice after cervical dislocation. Isopropanol was added to 50 mg liver tissue at a proportion of 20 g mL^−1^, and homogenized for 15 s followed by refrigeration overnight. Then, the mixture was centrifuged at 3000 rpm at 10 min. The supernatant was collected for subsequent arrays. Levels of lipid‐metabolism‐associated indicators, including TG, TC, HDL‐C, LDL‐C, FFA, and fasting blood glucose were tested with the kits purchased from the Nanjing Jiancheng Bioengineering Institute in China. Levels of endocrine indicators, including insulin (Immunodiagnostics, Hong Kong, China) and leptin (BioVendor, Brno, Czech Republic) were tested according to the instructions of the kits.

### Differentiation Experiment of Primary Preadiocytes

SVF cells were isolated from iWAT as previously reported.^[^ ^44^ ^]^ Briefly, 8‐week‐old mouse was sterilized and sacrificed by cervical dislocation. Subcutaneous iWAT was immediately dissected from the interscapular area under the skin. Isolated iWAT was minced on ice, followed by digestion with 10 mg mL^−1^ collagenase D (Roche, Switzerland) and 4 mg mL^−1^ Dispase II (Sigma, USA) for 40 min at 37 ℃. The homogenate was mixed well by pipetting and centrifugated at 700 × *g* for 10 min. Then, the deposit was subjected to a 70 µm strainer to collect SVF cell. Dissociative cells were plated on collagen (Sigma) coated plates supplemented with complete medium (DMEM [Sigma] containing 10% FBS [Sigma]). The induction was performed 2 days later via replacement to differentiation medium (complete medium with 125 µM Indomethacin [Sigma], 2 µg mL^−1^ Dexamethasone [Sigma], 0.5 mM 3‐Isobutyl‐1‐methylxanthine [Sigma], and 0.5 µM Rosiglitazone [Sigma]).

### RT‐qPCR

Total RNA was extracted from tissues and cells using the Trizol reagent (Invitrogen, USA) according to manufacturer instructions. Reverse transcription of the total RNA (2–5 µg) for each sample was performed using the cDNA reverse transcription kit (Accurate Biology, China). The gene expression levels in different samples were normalized to the *Hprt* mRNA expression.

### Statistical Analysis

Statistical analysis between two groups was conducted with unpaired Student's *t*‐test by GraphPad Prism 7.00 with *p* < 0.05 as statistically significant.

## Conflict of Interest

The authors declare no conflict of interest.

## Author Contributions

D.W., Q.M., X.Y., and X.C. contributed equally to this work. Y.H. and Y.P. are senior authors. Q.M., D.W., Y.H., and Y.P. conceived and designed this research. Q.M., X.Y., R.L., J.J., and D.W. performed the animal experiments. Q.M., X.Y., R.L., and Y.Z. conducted the cellular and molecular experiments. D.W., X.F., L.F., Y.C., and Q.M. performed the bioinformatics analysis of lipidomics and transcriptomics. Q.M., D.W., X.C., L.L., and X.Y. collected and analyzed the experimental data. D.W., Q.M., X.C., L.Y., K.S., P.Y., and J.J. wrote the draft. Y.H., N.L., Y.P., and X.C. reviewed and supervised the manuscript. N.L. oversaw the project. All authors have read and approved the published version of the manuscript. A few spelling mistakes were corrected on March 03, 2022.

## Supporting information

Supporting Information

## Data Availability

The data that support the findings of this study are available from the corresponding author upon reasonable request.
