# Supplementary material for: Microduplication of 16p11.2 locus Potentiates Hypertrophic Obesity in Association with Imbalanced Triglyceride Metabolism in White Adipose Tissue
Source: Mol Nutr Food Res. 2022 Feb 5;66(5):2100241. doi: 10.1002/mnfr.202100241 (PMC9286681; doi:10.1002/mnfr.202100241)
Supplement: Supplementary file 1 — Supporting Information [file MNFR-66-0-s001.pdf]

Supporting Information

Materials and Methods

1.1. Mice Genotyping

Genomic cDNA was extracted from mouse tails by tail buffers containing protease K (Sangon Biotech, China). The PCR system was configured according to the protocols provided by Jackson Laboratory. The primers used for detecting the 800 bp mutant band and the 324 bp internal positive control band were summarized in the Supporting Information Table S2.

1.2. Behavioral analysis

Open field test was assayed on 8- to 12-week-old male mice. The test was performed in the animal behavior room between 9:00 A.M. and 5:00 P.M. Mice were placed in a box (40 x 40 x 40 cm^3) and allowed to explore freely for 10 minutes. Traveling distance and velocity of movement were recorded and automatically analyzed by Noldus EthoVision XT10 (Noldus Information Technology; Leesburg, VA, USA).

1.3. Histopathological analyses

Sampling procedure Liver and WAT were collected after transcardiac perfusion of 4% PFA and post-fixed. Tissues were then subjected to embed with Tissue-Tek OCT (Sakura Seiki) for cryosectioning (20 μm) or with paraffin for paraffin-sectioning (3 μm). For cells staining, cells were incubated with 4% paraformaldehyde (PFA) for 30 min.

BODIPY (493/503) Staining Liver frozen sections or cells were incubated with diluted BODIPY (Thermo Scientific, USA) solution for 30 min in a dark room. After 3 times washes with PBST, the slides or cells were mounted with a fluoroshield mounting medium (Abcam, England) and imaged with a confocol laser scanning microscopy (LSM880, Zeiss, Germany).

H＆E staining Liver paraffin sections were subjected to H&E staining kit (Baso, China) according to the standard protocol and imaged with a BX53 microscope (Olympus, Japan).

Sudan III staining Liver frozen sections were stained with a Sudan III staining solution (Solarbio; China) according to standard protocols and imaged with a DM4B microscope (Leica, Germany).

Oil Red O staining Cells were subjected to Oil Red O staining kit procedures (Solarbio, China) according to standard protocols and imaged with a CKX53 microscope (Olympus, Japan).

Image quantifying The Image J (https://imagej.nih.gov/ij/) was used for quantifying the staining images. Image files were opened in the software and images were converted to 8-bit grayscale. The unit of length was set according to scale bars. 1) For measuring cell size: cells were manually outlined with the line tool of image J. 2) For measuring area and integrated density of fluorescent signal: after the optical density being corrected, the target fluorescent spots were distinguished from background via setting a certain threshold of grayscale. Then, the results could be automatically calculated and output by image J.

1.4. RNA Sequencing analysis

Twelve-week-old mice were randomly selected, including 4 male DP and 4 male WT mice. The epididymal white adipose tissue (eWAT) was dissected and immediately frozen in liquid nitrogen. RNA extraction and RNA Sequencing (RNAseq) analysis were performed by Applied Protein Technology (Shanghai, China). Briefly, total RNA was isolated using TRIzol (Thermo Fisher Scientific, Shanghai) and 2 mg RNA per sample was used as input material to prepare a cDNA library (paired end 250bp) for high-throughput sequencing with the HiSeq 2000 sequencing system (Illumina, Shanghai). After filtering low-quality and adapter sequences, reads were mapped to mouse genomes with Hisat2 software (Version 2.1.0) [2] with default settings. Read counts was subsequently extracted with the htseq-count script [3]. DESeq2 (Version 1.28.1) [4]

was used in differential gene discovery. GSEA [5] analysis was conducted to discover enriched pathways.

1.5. Lipidomic assay

Mice at the age of 12 weeks were randomly selected, including 6 male DP and 6 male WT mice. The eWAT was dissected and immediately frozen in liquid nitrogen. Firstly, lipids extraction was conducted. Briefly, 800 μL methyl tert-butyl ether and 240 μL pre-cooled methanol were added to each sample, followed by vortex mixing. The mixture was ultrasonic for 20 min at 4 ℃ and then centrifuged at 14000 g for 15min at 10 ℃. The upper organic phase was collected and dried under nitrogen. For mass spectrometry analysis, 200μL 90% isopropanol/acetonitrile added to obtain reconstitute. After centrifugation, the supernatant was collected for subsequent analysis. Applied Protein Technology (Shanghai, China) conducted the LC-MS/MS analysis using a 1μl supernatant for each sample with Q Exactive plus a mass spectrometer (Thermo Scientific, USA). The samples were separated with UHPLC Nexera LC-30A (SHIMADZU). Raw data processing, peak extraction, lipid identification, peak alignment, and quantification were conducted by LipidSearch[6] (version: 4.1, Thermo Scientific™).

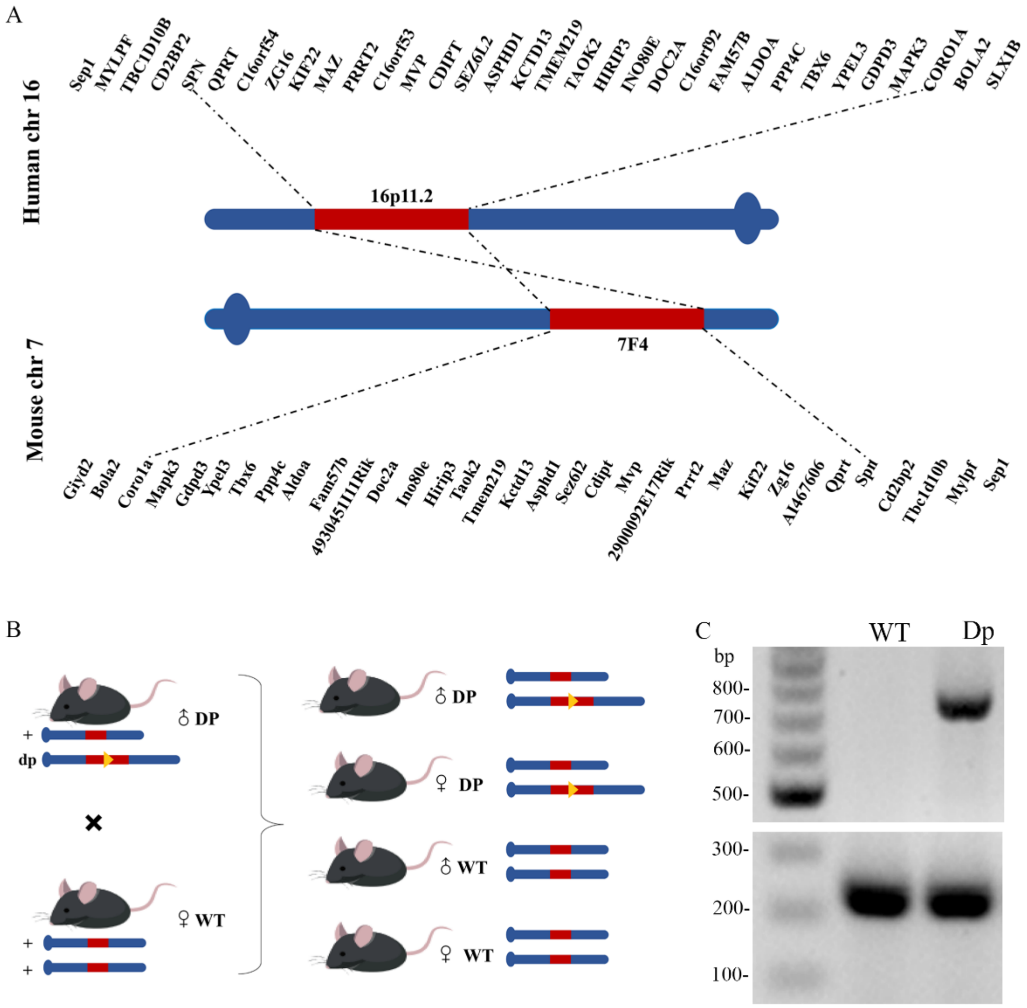

Supplementary Figure 1. Mouse model of 16p11.2 DP. (A) Genes mapping to the human 16p11.2 microduplication is conserved in the mouse. (B) Heterozygous male *dp/+* mice are crossed with female wild type mice to obtain heterozygous and WT genotype for subsequence

experiments. Yellow triangles represent *loxP* sites. (C) Molecular validation. The internal positive control band is 324bp, and the mutant band is about 800bp.

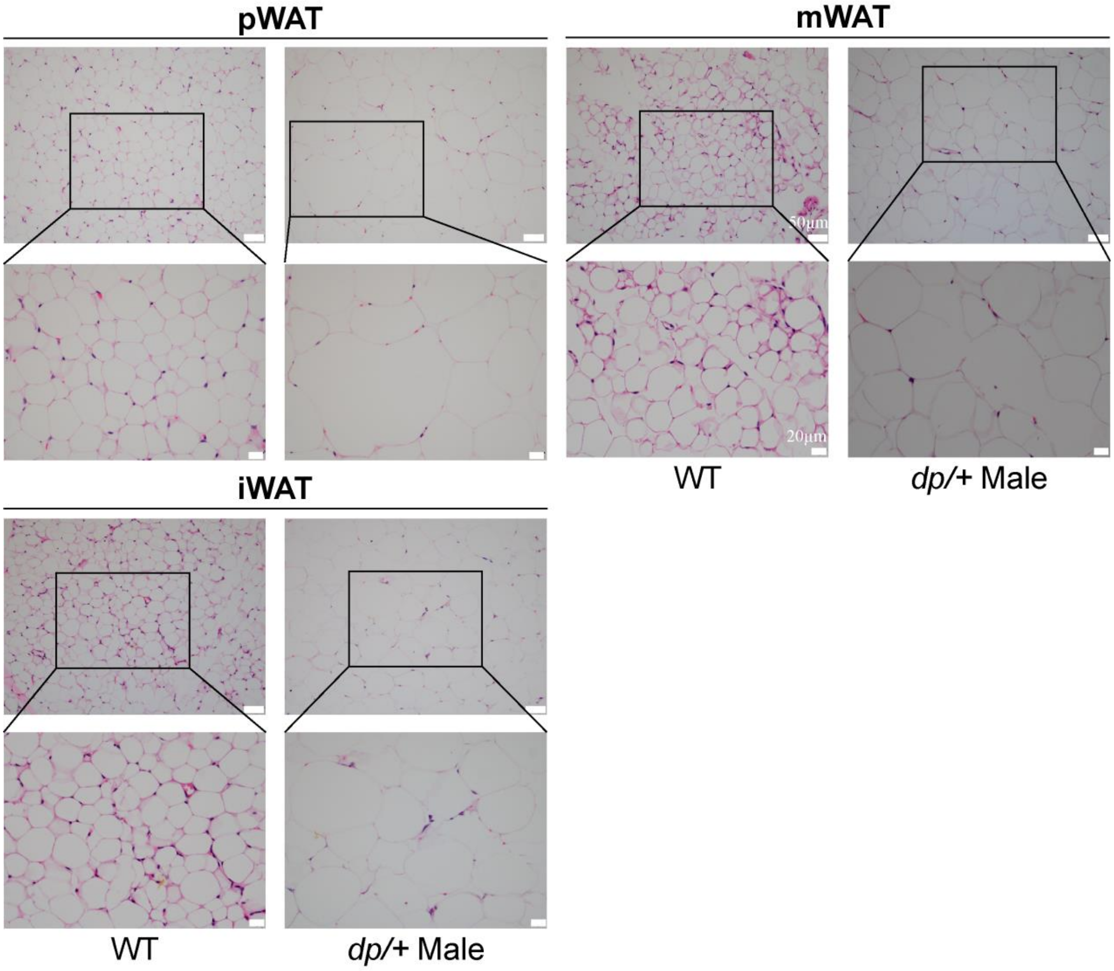

Supplementary Figure 2. Morphology of adipocytes in WAT. Representative H&E images of pWAT, mWAT and iWAT.

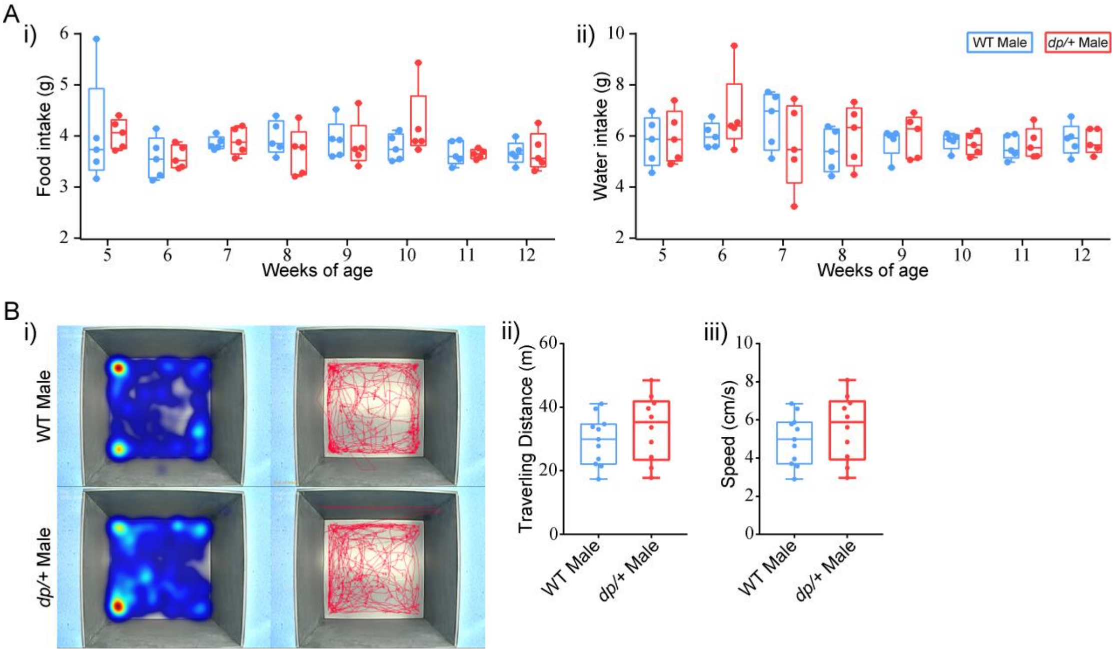

Supplementary Figure 3. Dietary and locomotive status of mice. (A) Statistical diagram for weekly i) food and ii) water intake from the 5th to 12th week after birth, WT n=5, *dp/+* n=5. (B) Open field test. i) The representative heat map and trajectory of the mice in 10 min. ii) Statistical diagram for i) travelling distance and ii) speed, WT n=11, *dp/+*, n=10.

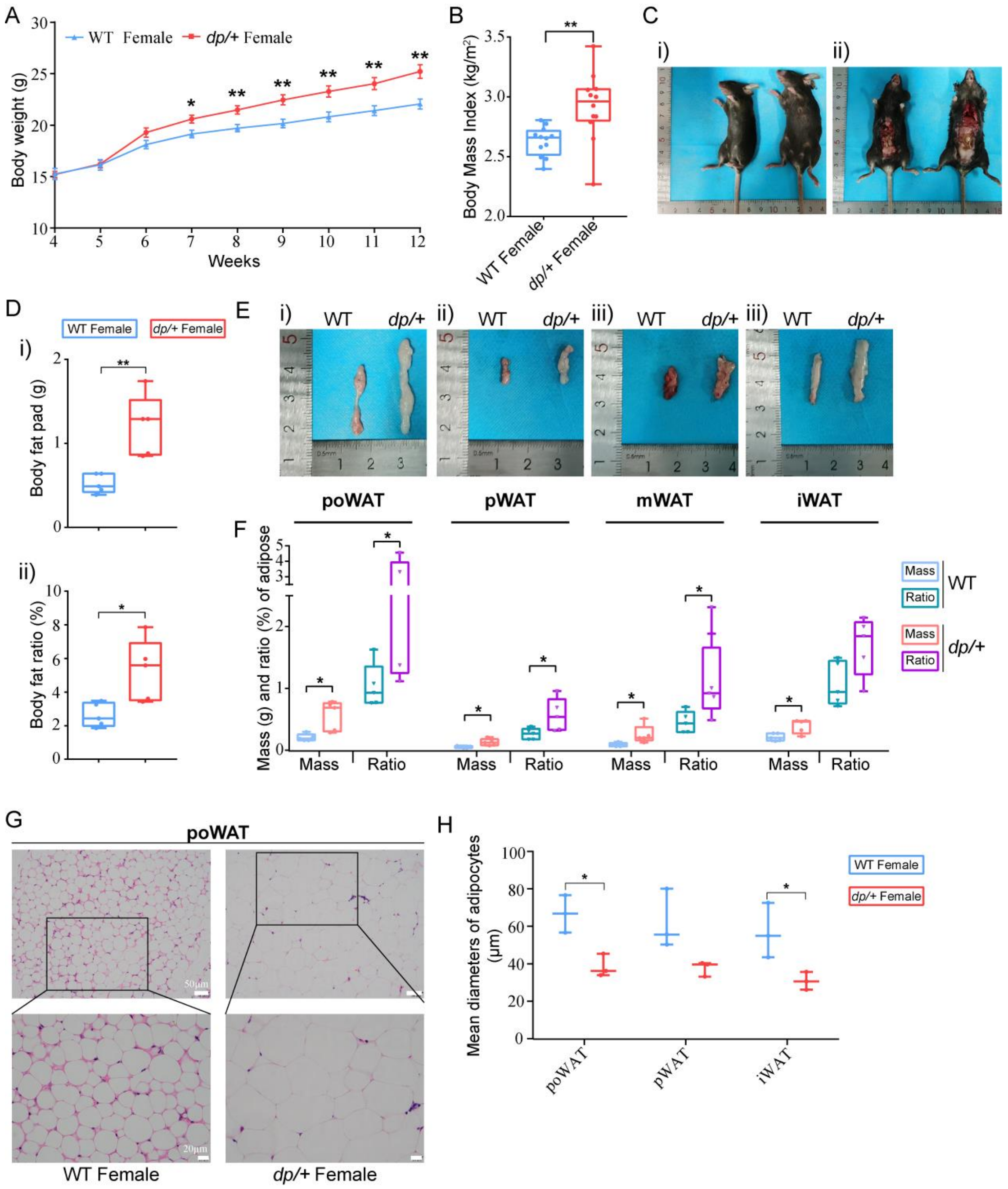

Supplementary Figure 4 Female *dp/+* mice exhibit obesity phenotypes on normal chow diet. (A) The curve for weight record of male mice from 4th to 12th week after birth and statistical analysis, WT n=8, *dp/+* n=8. (B) Statistical diagram for body mass index of male mice at 12-week age, WT n=12, *dp/+* n=12. (C) Representative images of female 12-week-old WT (left) and *dp/+* (right) mice. (D) Statistical diagram of fat pad mass (g) and fat ratio (%) of female 12-week-old mice, WT n=5, *dp/+* n=5. (E) Representative images of poWAT, pWAT, mWAT and iWAT from

female WT (left) and *dp/+* (right) mice at 12 weeks old. (F) Statistical diagram for adipose mass and ratio of eWAT, pWAT, iWAT and mWAT from female 12-week-old mice, WT n=5, *dp/+* n=5. (G) Representative H&E images of poWAT for female WT and *dp/+* mice at 12-week-old (Scale bars, 50μm, 20μm). (H) Statistical diagram for mean adipocytes diameters (μm) based on H&E staining images of poWAT, pWAT and iWAT, WT n=3, *dp/+* n=3. All data were presented as boxplots with whisker indicating the sample minimum to sample maximum through all the quartiles. Significance was evaluated by unpaired t-test between WT and *dp/+*, with $^{*}P < 0.05$， $^{**}P < 0.01$. WAT, white adipose tissue; poWAT, peri-ovarian WAT; pWAT, perirenal white adipose tissue; mWAT, mesenteric white adipose tissue; iWAT, inguinal white adipose tissue.

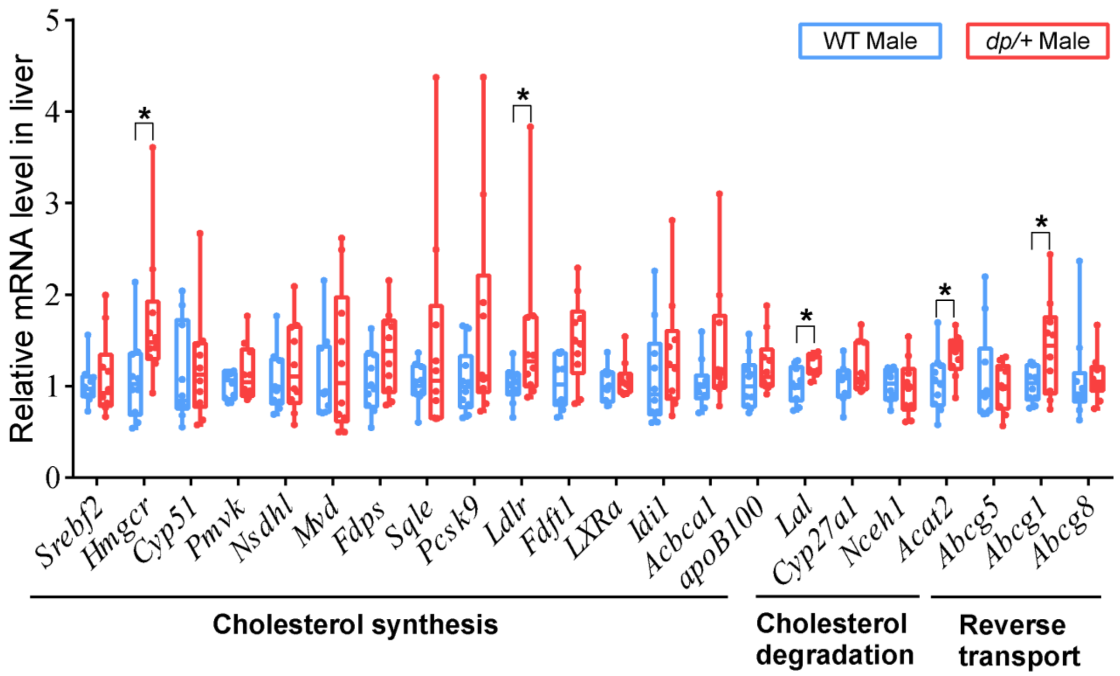

Supplementary Figure S5. The expression level of cholesterol-metabolism-associated genes in liver. Statistical diagram, WT n=10, *dp/+* =10. All data were presented as boxplots with whisker indicating the sample minimum to sample maximum through all the quartiles. Significance was evaluated by unpaired t-test between WT and dp/+, with *P < 0.05.

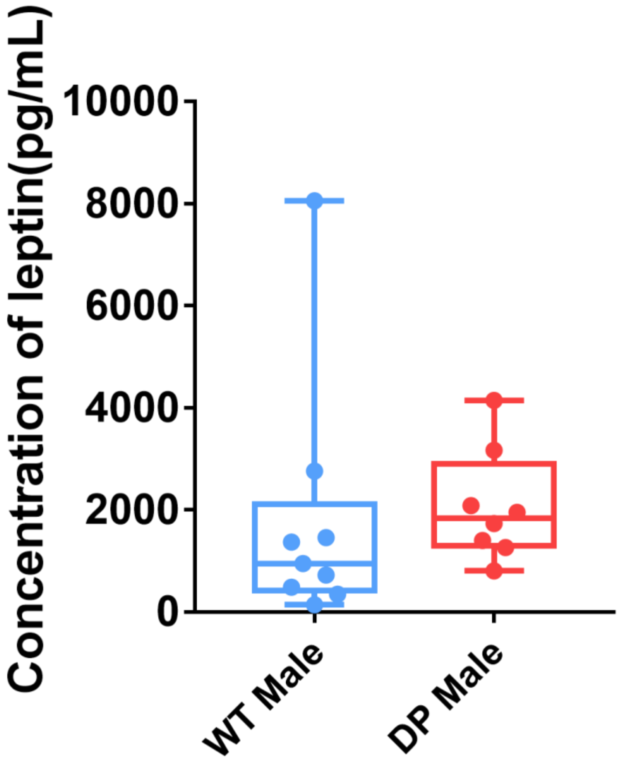

Supplementary Figure S6. The plasma level of leptin in mice. Statistic diagram, WT n=9, *dp/+* =8. All data were presented as boxplots with whisker indicating the sample minimum to sample maximum through all the quartiles. Significance was evaluated by unpaired t-test between WT and *dp/+*.

Supplementary Table 1 Triglyceride in muscle tissue for male WT and DP mice

Metabolic parameters (muscle)	Male (12 weeks)		
	**WT**	**DP**	***P* value**
Triglyceride, mmol/L	1.11±0.32	4.76±1.32	0.0149*

All data are presented as mean ± SEM, n=10. *$P < 0.05$，male WT mice versus male DP mice.

Supplementary Table 2 Primer for genotyping

Primer	Sequencing	Primer type	Reaction
15388	GGT AGA ATT TCG AGG TCG CTA G	Mutant Forward	B
15389	CAA GCT GAT CCG GAA CCC	Mutant Reverse	B
oIMR7338	CTA GGC CAC AGA ATT GAA AGA TCT	Internal Positive Control Forward	A
oIMR7339	GTA GGT GGA AAT TCT AGC ATC ATC C	Internal Positive Control Reverse	A

Supplementary Table 3 Primer of RT-qPCR

Gene	Forward primer	Reverse primer	product length
PPARγ	ATTAGATGACAGTGACTTGGC	TGTCTTGGATGTCCTCGATG	101 bp
C-EBPα	TCAGACCAGAAAGCTGAGTTGTG	TGGTCCCCGTGTCCTCCT	100 bp
Aacs	GCTGAGTTCTGGAAGTTCAG	CATCTGCAATCCCTTTGGATG	82 bp
Acly	TCAGTCCCAAGTCCAAGATCC	TCTCGGGAACACACGTAGTC	134 bp
Fasn	CCAAGCAGGCACACACAATG	AGTGTTCGTTCCTCGGAGTG	110 bp
Lipe	TGGAACTAAGTGGACGCAAG	TCAGACACACTCCTGCGCA	91 bp
Cidec	GGAAGGACATCAAGGGGGTG	TCACCTTCCAGCTTGACGAC	169bp
Srebf2	GCAGCAACGGGACCATTCT	CCCCATGACTAAGTCCTTCAACT	200 bp
Hmgcr	AGCTTGCCCGAATTGTATGTG	TCTGTTGTGAACCATGTGACTTC	104 bp
Cyp51	TGGAGCGAAAAGTCCACCAC	TGCATCACTCCCCAGAAGGTA	172 bp
Fdps	GGAGGTCCTAGAGTACAATGCC	AAGCCTGGAGCAGTTCTACAC	155 bp
Mvd	ATGGCCTCAGAAAAGCCTCAG	TGGTCGTTTTTAGCTGGTCCT	157 bp
Nsdhl	ACGCCATGAAGCCTATTGACT	GGTCCCTTGGGCCGAAAAT	135 bp
Pmvk	CTTGGAGGTAACATCTGTGCTC	GTGCTCGCATCCAGAAGTCTC	104 bp
Fdft1	GTTTGAAGACCCCATAGTTGGTG	CACATCTACGTTCTCTGGCTTAG	190 bp
Sqle	AGTTCGCTGCCTTCTCGGATA	GCTCCTGTTAATGTCGTTTCTGA	148 bp
Pcsk9	TTGCCCCATGTGGAGTACATT	GGGAGCGGTCTTCCTCTGT	112 bp
Ldlr	AGTGGCCCCGAATCATTGAC	CTAACTAAACACCAGACAGAGGC	107 bp
Abca1	GCTTGTTGGCCTCAGTTAAGG	GTAGCTCAGGCGTACAGAGAT	135 bp

Idi1	TGGGGCTGACACCAAGAAAA	CAGCTCGCCTGGGTTACTTA	196 bp
LXRa	CTCAATGCCTGATGTTTCTCCT	TCCAACCCTATCCCTAAAGCAA	150 bp
apoB100	TTGGCAAACTGCATAGCATCC	TCAAATTGGGACTCTCCTTTAGC	142 bp
Lal	TGTTCGTTTTCACCATTGGGA	CGCATGATTATCTCGGTCACA	103 bp
Cyp27a1	CCAGGCACAGGAGAGTACG	GGGCAAGTGCAGCACATAG	139 bp
Acat2	CCTGTCTCTCGGTTTCCCAT	GCTGCCCACCTTTCAACAAT	164 bp
Nceh1	TTGAATACAGGCTAGTCCCACA	CAACGTAGGTAAACTGTTGTCCC	189 bp
Abcg1	GTGGATGAGGTTGAGACAGACC	CCTCGGGTACAGAGTAGGAAAG	145 bp
Abcg5	CCAGATTATGTGCATCTTAGGCA	CTGCTCAGAAAAACGTCGCT	186 bp
Abcg8	CTGTGGAATGGGACTGTACTTC	GTTGGACTGACCACTGTAGGT	108 bp
G0s2	GCCACCGAATCCAGAACTGA	TTGATTGCTCGCACAGCCTA	108 bp
Plin4	GTGTCCACCAACTCACAGATG	GGACCATTCCTTTTGCAGCAT	160 bp
Plin5	CAGAGCAAACACCGTACCCAG	GGGATGGAAAGTAGGGCTAGG	105 bp
Pnpla2	CAACGCCACTCACATCTACGG	GGACACCTCAATAATGTTGGCAC	106 bp
Hprt	TGCTGACCTGCTGGATTACA	TTTATGTCCCCCGTTGACTGA	120 bp

[1] Lim, S., Honek, J., Xue, Y., Seki, T., Cao, Z., Andersson, P., Yang, X., Hosaka, K., Cao, Y., Cold-induced activation of brown adipose tissue and adipose angiogenesis in mice. *Nature protocols* 2012, *7*, 606-615.

[2] Kim, D., Langmead, B., Salzberg, S. L., HISAT: a fast spliced aligner with low memory requirements. *Nat Methods* 2015, *12*, 357-360.

[3] Anders, S., Pyl, P. T., Huber, W., HTSeq--a Python framework to work with high-throughput sequencing data. *Bioinformatics* 2015, *31*, 166-169.

[4] Love, M. I., Huber, W., Anders, S., Moderated estimation of fold change and dispersion for RNA-seq data with DESeq2. *Genome Biol* 2014, *15*, 550.

[5] Subramanian, A., Tamayo, P., Mootha, V. K., Mukherjee, S., Ebert, B. L., Gillette, M. A., Paulovich, A., Pomeroy, S. L., Golub, T. R., Lander, E. S., Mesirov, J. P., Gene set enrichment analysis: a knowledge-based approach for interpreting genome-wide expression profiles. *Proc Natl Acad Sci U S A* 2005, *102*, 15545-15550.

[6] Taguchi, R., Ishikawa, M., Precise and global identification of phospholipid molecular species by an Orbitrap mass spectrometer and automated search engine Lipid Search. *J Chromatogr A* 2010, *1217*, 4229-4239.
